# Wavelength-shifting properties of luminescence nanoparticles for high energy particle detection and specific physics process observation

**DOI:** 10.1038/s41598-018-28741-y

**Published:** 2018-07-12

**Authors:** Sunil Sahi, Stephen Magill, Lun Ma, Junqi Xie, Wei Chen, Benjamin Jones, David Nygren

**Affiliations:** 1 Department of Physics, University of Texas at Arlington, Arlington, TX 76019 USA; 2 Argonne National Laboratory, Argonne, IL 60439 USA

## Abstract

Ultraviolet (UV) photon detection is becoming increasingly important in the quest to understand the fundamental building blocks of our universe. Basic properties of neutrinos and Dark Matter are currently being explored through interactions with noble elements. In response to interactions with fundamental particles, these elements emit scintillation photons in the UV range. However, most available detectors have poor response in the UV so it is typically necessary to shift UV to a wavelength, matching the sensitivity of the viable detectors. We report on development of UV-enhanced photosensors using wavelength-shifting properties of nanoparticles. Several nanoparticle coatings were tested for absorption of UV light with subsequent emission in the visible wavelength for high energy particle detection. ZnS:Mn,Eu, ZnS:Mn, CuCy (Copper Cysteamine) and CdTe nanoparticles all exhibited enhanced detection for wavelengths in the range 200–320 nm in several different tests, while ZnS:Ag and CdS nanoparticle showed little or no enhancement in that range. In addition, various LaF_3_:Ce nanoparticle concentrations in approximately constant thickness of 2,5-diphenyloxazole (PPO)/polystyrene bases were also tested to optimize the nanoparticle concentration for the best outcome. Our studies indicated that ZnS:Mn,Eu, ZnS:Mn, Cu-Cy, CdTe and LaF_3_:Ce nanoparticles show potential for light detection from fundamental particle interactions.

## Introduction

Ultraviolet (UV) photon detection is becoming increasingly important in our quest to understand the fundamental building blocks of our universe. Basic properties of neutrinos and Dark Matter are currently being investigated through interactions with noble elements. Large liquid argon TPC (time projection chamber) detectors exposed to accelerator neutrino beams are being developed for the study of neutrino oscillations at short^1,2^ and long^3^ baselines. Argon detectors are also used in studies of the recently observed low-energy coherent neutrino-nucleus scattering process^4^. Sensitive dark matter searches using liquid argon^5–8^ and xenon^9–11^ have been deployed, and larger detectors still are planned^12^. Both liquid^13^ and gas^14^ phase detectors using ^136^Xe as an active medium are used to search for the ultra-rare process of neutrinoless double beta decay. Thus, noble element TPC detectors represent a versatile and indispensable tool for modern particle physics research. Energy loss by charged particles in noble liquids and gases generate eximers which decay radiatively, emitting scintillation photons in the UV wavelength range. Also, many crystal scintillators, some with multiple wavelength emissions, emit a component of light in the UV wavelength range^15^. In the latter case, the fast timing of the UV emission is also useful, particularly in high rate experiments such as the muon to electron (Mu2e) conversion experiment^16^ where large background rates must be separated from possible signal interactions.

Past experiments have used chemical wavelength-shifters, e.g., 1,1,4,4 Tetraphenyl Butadiene (TPB)^17^, to absorb UV light and re-emit it in the visible wavelength range where existing photosensors have good sensitivity. TPB coatings have several drawbacks, including their instability in noble environments^18^, photo-degradation behavior^19^, and limited Stokes shift, which causes re-absorption of emitted light^20^. Many nanoparticles have also exhibited wavelength-shifting properties that could make them attractive options for UV photon detection, effectively extending the useful wavelength detection range of existing visible-sensitive photosensors.

Wavelength shifting properties of nanoparticles can be understood in terms of the atomic properties of the materials when their dimensions are measured in a few to 100 nm range^21^. When the dimensions of a particle (atoms or molecules) are smaller than the electron-hole distance (Bohr exciton radius) in that material, some particle properties are changed in very significant ways^21–24^. For example, the energy band gap is increased and many discrete energy levels form at the edges of the band gap^21–24^. So, the absorption of photons is shifted to higher energies (from visible to UV wavelengths). Absorption of a photon puts the molecule in an excited state, which then relaxes back to its ground state by emission of a longer wavelength photon^25^. The difference in wavelength between the excitation spectrum (absorbed photon wavelength) and the emitted spectrum is called the Stokes shift. By this mechanism, the absorption of a high energy photon is determined by detection of the Stokes-shifted emitted photon – the nanoparticle effectively operates as a wavelength-shifter. In addition, the emission wavelength can be tuned since the energy gap is highly dependent on the nanoparticle size^21–24^. These changes in particle properties occurring for particle sizes at the Bohr exciton radius are collectively known as quantum confinement effects^21–24^. Since these effects are happening at the atomic or molecular level, absorption and emission of light is more efficient for nanoparticles than it is in bulk materials as a result of quantum size confinement, mainly due to the increase of the electron-hole wavefunction overlap and the split of the energy levels into discreet values^21–24^. Also, since the size of the nanoparticles determines the wavelength range of the shifted light, a nanoparticle wavelength-shifter can be tuned to match the optimal sensitivity of an existing photodetector.

The quantum size confinement is not only effective for undoped semiconductors but also applicable for doped semiconductors^21,26^. For doped nanomaterials, the emission wavelength is mainly determined by the luminescence centers. However, the emission efficiency can be modified by size effects^21^. Just like undoped semiconductor quantum dots, the luminescence of doped nanoparticles is also enhanced due to quantum size confinement as a results of the increase in the electron-hole wavefunction overlap of trapped excitons^27^.

An optimal photodetector for a specific physics process would then consist of a type-selected nanoparticle coating or infusion in a substrate, with the nanoparticle size set so that the Stokes-shifted emitted light would match the peak sensitivity of a chosen photodetector. In a previous publication^28^, it was shown that Si nanoparticles exhibited wavelength-shifting properties - absorbing UV light and subsequently emitting light in the visible wavelength range. Due to the effects of quantum size confinement, the band gap of Si in nanoparticle form is increased to over 3 eV, which can be compared to its normal value in elemental form of 1.1 eV. This changes the photon absorption properties of the element, enhancing its response to wavelengths in the UV range^29^.

In an ongoing effort to find suitable nanoparticle candidates for use in applications requiring detection of UV light, several luminescent nanoparticle samples were prepared at the University of Texas at Arlington for initial testing. Here, we have selected several nanoparticles, namely ZnS:Mn, Eu^30^, ZnS:Mn^31,32^, CuCy (Copper cysteamine)^33,34^, CdTe^35,36^, CdS^37,38^, ZnS:Ag^39^, and LaF_3_:Ce^40^, which are known to emit intensive photoluminescence by UV excitations. We have also successfully demonstrated that different nanoparticles can be combined using the principle of Fluorescence Resonance Energy Transfer (FRET) to absorb UV light and emit the visible light as reported previously^36,41–43^. The goals of this work are to identify various nanoparticle types according to their different sensitivities to UV wavelengths, thus optimizing the nanoparticle type for a wavelength-shifting coating. In addition, studies of the chosen nanoparticle size variations would lead to optimization of the nanoparticle–photosensor combination, resulting in a tuned photodetection system for a specific physics process observation.

## Results

### The nanoparticles

**Figure 1:**
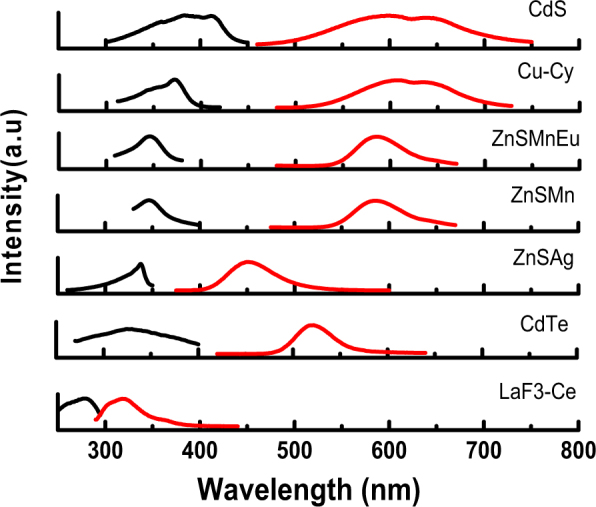
The excitation (black) and emission (red) spectra of CdS, Cu-Cy, ZnS:Mn,Eu, ZnS:Mn, ZnS:Ag, CdTe, and La_x_Ce_1−x_F_3_ (0 ≤ x < 1).

**Figure 2:**
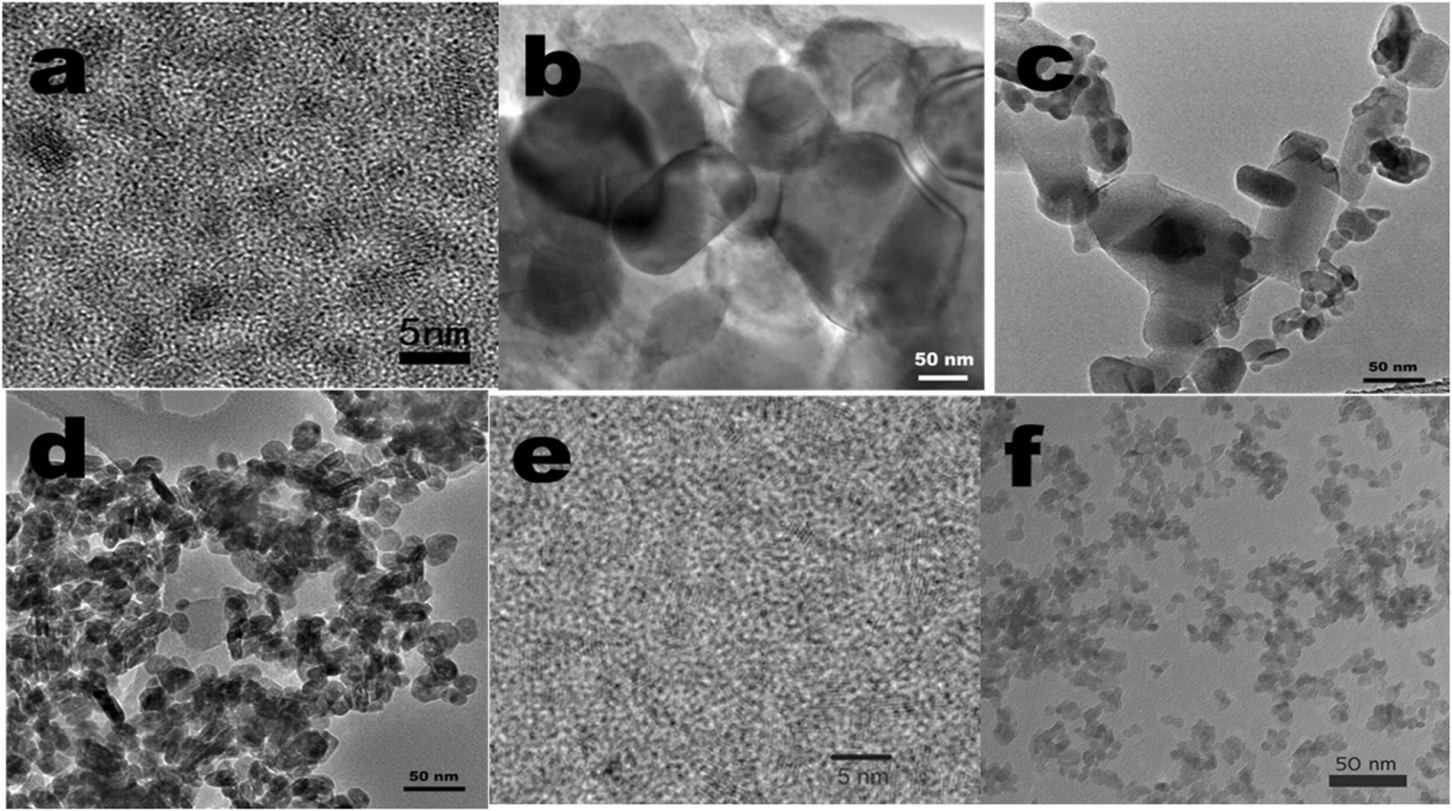
TEM images of (**a**) CdS, (**b**) Cu-Cy, (**c**) ZnS:Mn,Eu, (**d**) ZnS:Mn, (**e**) CdTe, and (**f**) La_x_Ce_1−x_F_3_ (0 ≤ x < 1).

For these tests, several different nanoparticle sample coatings were prepared based on their previously-identified optical properties. They are identified by their chemical composition as: ZnS:Mn,Eu, ZnS:Mn, CuCy, CdTe, ZnS:Ag, La_x_Ce_1−x_F_3_ (0 ≤ x < 1) and CdS. These nanoparticles have strong absorption in the UV region and emission in the visible region as reported in the previous studies which make them suitable for application as wavelength shifting materials. Figure 1 shows the photoluminescence spectra of different nanoparticles and their excitation and emission wavelengths are summarized in Table 1. Figure 2 shows the transmission electron microscope (TEM) images of the nanoparticles and their mean sizes are listed in Table 1.

**Table 1:** The excitation, emission peak wavelengths and decay lifetimes of tested nanoparticles.

Properties	CdS	Cu-Cy	ZnS:Mn,Eu	ZnS:Mn	ZnS:Ag	CdTe	La_x_Ce_1−x_F_3_
Excitation (nm)	410	370	340	340	340	330	247
Emission (nm)	600	608	585	585	450	530	330
Lifetime (ns)	240^44^	6190^45^	80-192^46^	180000^47,48^	7600^49^	32^50^	22^51^
Size (~nm)	4	150	60	20	500	2	10

As we see in Fig. 1, except for LaF_3_:Ce that shows the excitation spectrum extended to 200 nm while the others did not show excitation at the 200–300 nm range. The excitation spectra are different from the absorption spectra and all the samples have absorption extended to 200 nm or even shorter. So, the excitation at 200–300 nm range can produce luminescence. As for this particular application, we are more interested in the enhanced response shorter than 300 nm, so our measurements were mainly for the range of 200–300 nm.

Also, doping can effectively impact the luminescence behaviors. When Eu^2+^ is doped into ZnS:Mn, it increased the luminescence intensity of Mn^2+^ emission but it did not change the emission position as in both ZnS:Mn,Eu and ZnS:Mn, the emission is from Mn^2+^ as discussed in our previous paper^30^. The luminescence enhancement is due to the energy transfer from Eu^2+^ to Mn^2+^ which has been detailed in our previous publication^30^.

### Baseline response of photosensor with no nanoparticle enhancement

**Figure 3:**
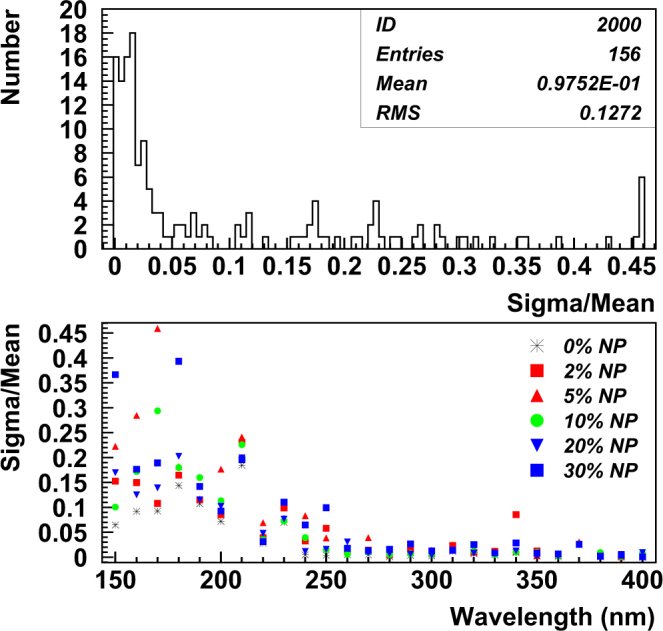
(top) Histogrammed values of Sigma/Mean for all La_x_Ce_1−x_F_3_ nanoparticle sample data, (bottom) Sigma/Mean versus wavelength for all samples.

Control data was collected by testing the sensitivity of samples with no nanoparticles in the same configurations and techniques as the nanoparticle tests were done. A scanning monochromator with D_2_ lamp was used to illuminate the samples with a wavelength range starting as low as 150 nm and increasing in steps of 10 nm (or 5 nm in some cases) to a maximum value of 400 nm. This technique resulted in coverage of the wavelength range defining the turn-on of sensitivity of the MPPCs (Multi-Pixel Photon Counters). Due to the presence of large fluctuations at wavelengths <200 nm, results of the scans will be reported here for wavelengths >190 nm. Figure 3 shows all values of Sigma/Mean for the data taken with different nanoparticle concentrations both histogrammed and as a function of scanning wavelength.

**Figure 4:**
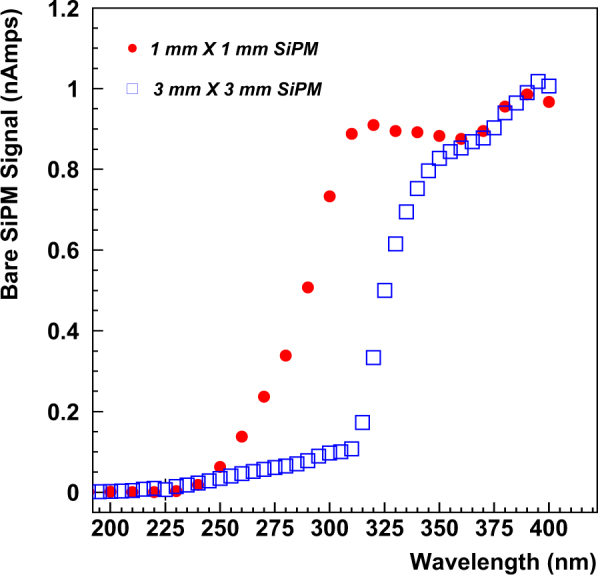
Baseline response of 1 mm × 1 mm (red dot), and 3 mm × 3 mm (blue square) MPPCs in the wavelength range from 195 to 400 nm.

For the tests of nanoparticles deposited directly on the surface of the MPPCs, response of an uncoated MPPC was used to compare to the coated ones. The uncoated MPPCs were selected to have operating characteristics (as supplied by the manufacturer) very similar to the coated ones. Figure 4 shows the baseline response to the monochromator scan of the uncoated 1 mm × 1 mm MPPC and the 3 mm × 3 mm MPPC. The spot size of the D2 lamp illumination on the MPPCs was set using the 3 mm × 3 mm MPPC. The (round) shape of the lamp spot after focusing and collimation was approximately 1 mm in diameter, so it would fit entirely within the area of the 3 mm × 3 mm MPPC but was slightly greater than the area of the 1 mm × 1 mm MPPC. The baseline signals obtained roughly corresponded to the areas of exposure on the 2 MPPC sensors.

After the data points were normalized to the exposure area of the MPPCs, they show very similar response for wavelengths greater than ~350 nm as expected. Neither of the MPPCs were sensitive at all to light with wavelengths less than ~250 nm. The 3 mm × 3 mm MPPCs are only sensitive to wavelengths greater than ~320 nm, while the response of the 1 mm × 1 mm MPPC turns on ~60 nm lower in wavelength at ~260 nm.

**Figure 5:**
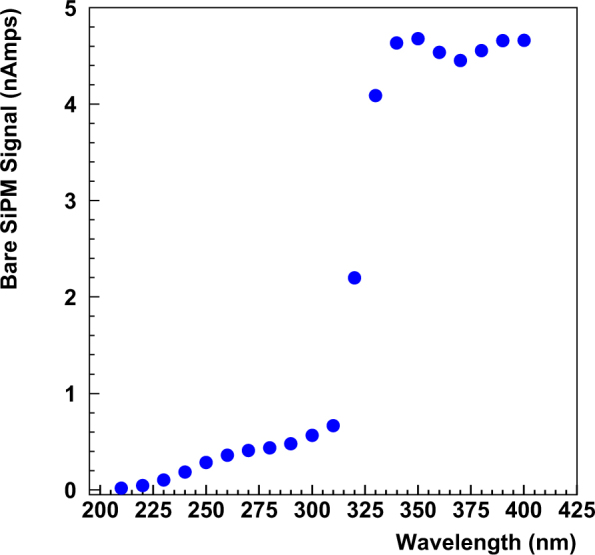
Response of 3 mm × 3 mm MPPC to reflected light from uncoated plastic tape.

For the measurements of nanoparticle samples deposited on the tape, the baseline response was measured from an area of the tape not coated with nanoparticles. Figure 5 shows the response of the MPPC for a region of uncoated tape. This measurement was taken as backward emission of light from the uncoated tape in the same way as the coated measurements. As confirmation of the method, no reflected or backward emitted light was seen when the tape was irradiated with UV light from a handheld UV light source.

For the case of the polystyrene buttons, a button was prepared with 0% (by weight) nanoparticles and was used as the baseline for these tests. The response of the MPPC for a monochromator scan on this button was essentially the same function of wavelength as shown in Fig. 5 for the 3 mm × 3 mm MPPC. No response below the standard MPPC sensitivity range was seen in this test. Also, since this response is nearly identical to the response of the MPPC to light reflected from the plastic tape, this indicates that no sensitivity to low wavelength photons was observed due to the presence of PPO in the transparent button.

These baseline scans clearly show that all of the MPPC testing configurations used in these measurements were not directly sensitive to light with wavelengths less than ~260 nm for the 1 mm × 1 mm MPPC and less than ~320 nm for the 3 mm × 3 mm MPPCs.

## Discussion

Light beam alignment from the D_2_ lamp to the MPPC was eliminated as a systematic effect by fixing the MPPC with respect to the light beam spot. The beam spot size was forced by focusing and collimation to be ~1 mm in diameter and the MPPC was aligned so that the beam spot was centered on the MPPC active area. No stray light could enter the MPPC from either outside the light-tight enclosure or scattered light from the D_2_ lamp.

The stability of response was determined by repeating measurements immediately after the original tests and also several days after the initial trials. Variation in the response of a 3 mm × 3 mm MPPC with no nanoparticle coating was found to be less than 2% in the wavelength range of 200–400 nm with response to wavelengths less than ~320 nm near zero. The variation in the 1 mm × 1 mm MPPC was also found to be less than 2%. For the backwards emitted response measurements in the tested wavelength range, variation in the response for conditions with no nanoparticle deposition was also found to be less than 2% (using the 3 mm × 3 mm MPPC for these tests). Finally, for the measurements of nanoparticle concentrations in polystyrene buttons, the variation in response of the MPPC covered with a button with no nanoparticles was also less than 2%, again using the 3 m × 3 mm MPPC.

**Figure 6:**
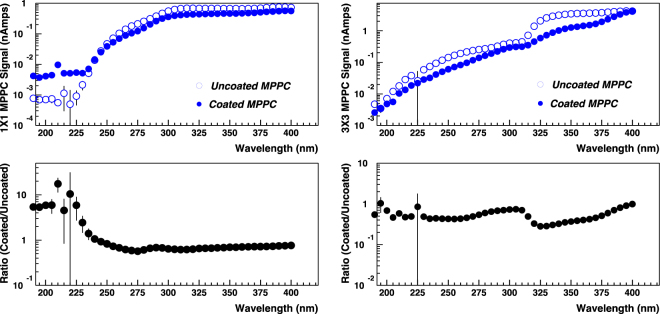
Response of Coated (blue dots) and Uncoated (blue circles) sensors for (top left) 1 mm × 1 mm and (top right) 3 mm × 3 mm MPPCs and corresponding ratios of Coated to Uncoated response (bottom).

**Figure 7:**
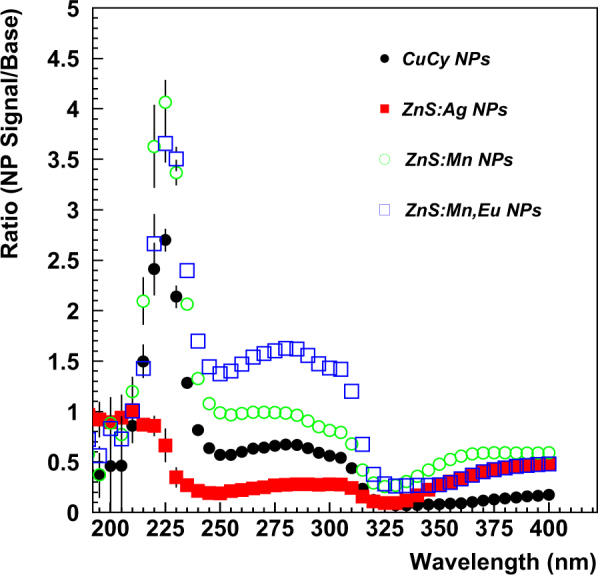
Ratio of nanoparticle to no nanoparticle response for 4 samples deposited on transparent tape.

**Figure 8:**
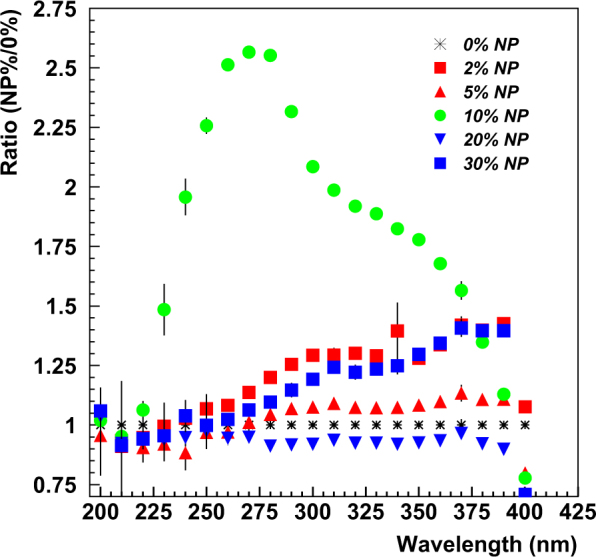
Ratio of responses versus wavelength for 6 sample buttons with various concentrations of LaF_3_:Ce nanoparticles from 2 wt% to 30 wt% (black stars are the 0 wt% ratio).

The results from tests of MPPCs with nanoparticle coatings applied directly to the protective resin on the face of the sensor are shown in Fig. 6. The 1 mm × 1 mm MPPC was coated with CdTe nanoparticles and showed an enhanced response compared to an uncoated MPPC in the wavelength range of ~200 nm to 240 nm. For the 3 mm × 3 mm MPPC, coated with CdS nanoparticles, no enhanced response was seen. Because the thickness and transparency of the coating was not completely controlled, it is possible that the coating on the 3 mm × 3 mm MPPC was more opaque than that on the 1 mm × 1 mm MPPC, resulting in no transmission of any emitted light. We plan to test these nanoparticles again with more control over the coating thickness and the nanoparticle concentration, which may affect the transparency of the sample. Figure 7 shows the results of backwards emission from nanoparticles deposited on the transparent tape. Three of the 4 samples show significant enhancement, especially in a narrow wavelength range of ~200 to 250 nm. We will investigate these responses further–having a peaked response is very desirable, for example, to detect fast scintillator light response from a crystal in a particular narrow wavelength range with reduced response in other undesirable wavelength regions. Figure 8 shows the results from transparent plastic buttons of constant thickness infused with various concentrations of LaF_3_:Ce nanoparticles. The largest enhancement was measured for a LaF_3_:Ce nanoparticle concentration of 10% in PPO/Polystyrene base. The enhanced response has a broad peak from ~240 nm to ~390 nm, making it a good candidate for near-UV applications.

To summarize, enhancement of the wavelength response of a 1 mm × 1 mm Hamamatsu MPPC was observed in the wavelength range of 200–240 nm for CdTe nanoparticles deposited directly on the surface of the photosensor when compared to an uncoated MPPC. CdS nanoparticles deposited in a similar manner on a 3 mm × 3 mm MPPC exhibited little or no response in the tested wavelength range of 200–400 nm. For the backward-emitted light from nanoparticle coatings deposited on plastic tape, 3 of the 4 tested nanoparticle samples (ZnS:Mn,Eu, ZnS:Mn, Cu-Cy) exhibited strong enhancement in the range of 200–250 nm. ZnS:Ag nanoparticles showed little enhancement in the tested wavelength range. In tests of variable LaF3:Ce nanoparticle concentrations in constant thickness ~transparent samples, the sample button containing a nanoparticle concentration of 10% showed the best enhanced response over a large wavelength range of 225–375 nm. It appears that for these tested samples, the 10% sample produced an optimal emission-absorption response. Our studies indicate that ZnS:Mn,Eu, ZnS:Mn, Cu-Cy, CdTe and LaF_3_:Ce nanoparticles have good potential for particle detection to uncover the building blocks of our universe.

## Materials and Methods

### Preparation of the nanoparticles

The nanoparticles were prepared by methods described in our previous publications ZnS:Mn,Eu^30^, ZnS:Mn^31,32^, CuCy (Copper cysteamine)^33,34^, CdTe^35,36^, CdS^37,38^, ZnS:Ag^39^ and LaF3:Ce^40^. Generally, we used two methods to prepare these luminescence nanomaterials: wet chemistry and solid-state reaction. For example, the ZnS:Mn nanoparticles were prepared using a similar solid-state diffusion method^30^. Briefly, 1.94 g ZnS and 28.6 mg MnCl_2_ were ground together thoroughly and covered with carbon charcoal in a crucible. The crucible was sintered at 800 °C for 3 h before being cooled to room temperature.

CdTe and CdS nanoparticles were synthesized in aqueous solutions using 3-mercaptopropionic acid (3MPA) as a stabilizing agent. As an example, CdS nanoparticles were synthesized as fellows: Briefly, 0.5 millimole cadmium chloride was dissolved in 50 ml DI water followed by the addition of 110 µl 3-mercaptopropionic acid under vigorous stirring. The pH of the solution was changed to 10 by using 1 M NaOH aqueous solution. Finally, 0.5 millimole thiourea in 10 ml water was added dropwise and reflux at 90 °C for 3.5 hrs. The final product was cooled down to room temperature and the solvent was evaporated using a rotary evaporator. The precipitate was washed with a water-acetone mixture for 3 times and dispersed in 20 ml of de-ionized (DI) water. The as synthesized CdS nanoparticles were highly dispersible in DI water.

### Preparation of nanoparticle samples for testing

The CdTe and CdS nanoparticles were deposited in solution on the surface resin coating of a Hamamatsu MPPC. CdTe nanoparticles were deposited on a 1 mm × 1 mm square MPPC and CdS nanoparticles were deposited on a larger 3 mm × 3 mm MPPC. The droplets of nanoparticles in solution were deposited on the surface of the MPPC and the liquid was allowed to evaporate, leaving a dry nanoparticle deposition. For these tests, control of the uniformity of nanoparticle concentration over the surface of the MPPC was not attempted. It was noticed that surface tension effects tended to sweep the nanoparticles to the edges of the surface as the liquid solution evaporated, resulting in a non-uniform distribution of the nanoparticles over the resin surface. Nevertheless, tests were performed on these devices using a similar setup as used in our earlier publication^28^ – in this case no plastic film was needed since the nanoparticles were deposited directly on the MPPC resin surface. The resin coating acted as the plastic film, effectively blocking photons of wavelength less than ~320 nm from impacting on the silicon of the MPPC.

Nanoparticle samples of ZnS:Mn,Eu, ZnS:Mn, CuCy, and ZnS:Ag were deposited on clear plastic tape in ~3 mm × ~3 mm square areas. The thickness and light transmission of each of these samples was such that the samples were opaque to visible and UV light. In our standard setup for testing, looking for light to be transmitted through the samples, no light was seen. Since these samples showed clear luminescence in the presence of a handheld UV light, a slightly different test was done with the monochromator setup. Instead of placing the MPPC behind the samples to collect transmitted light, the detector was positioned in front of and off to one side of the samples. In this way, emitted light at angles backwards to the direction of the incoming photons and also any reflected light was detected by the MPPC.

Finally, samples were prepared in which the visible light transmission through the sample was kept high by varying the concentration of nanoparticles in a transparent medium. Samples of La_x_Ce_1−x_F_3_ nanoparticles in PPO/polystyrene buttons were prepared as discussed in a previous work^44^. The thickness of the button was kept approximately constant while varying the nanoparticle concentration from 0% to 30% by weight (0%, 2%, 5%, 10%, 20%, and 30% concentrations) in the polystyrene base which also included a small amount (1%) of 2,5-diphenyloxazole (PPO). These samples were designed to determine if an optimal nanoparticle concentration would emerge, while keeping the base material transparent enough to transmit any emitted visible light.

These tests were all designed to test absorption of the nanoparticles with at least some emission of absorbed light in the visible range seen by a photosensor with no sensitivity for wavelengths less than ~320 nm. No attempt was made to tune the nanoparticle sizes to get an optimal emitted wavelength for the MPPC photosensor. Future tests will be done comparing the results of like nanoparticles of different sizes. The optimal wavelength sensitivity of the MPPC used in these tests is characterized by a broad peak centered at ~425 nm. For those samples for which no enhancement was observed, it may be that there was indeed enhancement, but the emitted light was not detected by the photosensor – this will be investigated in future tests in which the emitted wavelength spectrum will be determined using a spectrophotometer device.

### Experimental set up for emission measurement

The experimental setup consisted of a scanning monochromator equipped with a Deuterium lamp/fused silica window combination which produced light with wavelengths >150 nm. Transmission of UV light from the lamp to the test setup was done inside a black box in the room air atmosphere. This resulted in light measurements which sometimes were dominated by large fluctuations, especially at wavelengths <200 nm or so. In the future, we will control this by enclosing the entire setup in a N_2_ atmosphere, reducing the signal fluctuations at low wavelengths. Lenses and a collimator system were focused and formed the light into an approximately 1 mm diameter spot: (1) at the surface of the MPPC, which has an active area of 1 mm^2^ (9 mm^2^) for the 1 mm × 1 mm (3 mm × 3 mm) directly coated MPPCs, (2) into a similar 1 mm diameter spot centered on the nanoparticle coatings on tape, each of which had an area of ~9 mm^2^, and (3) also the same spot size centered on one of the polystyrene buttons which was then placed directly in front of the MPPC. The MPPC was not powered with a bias voltage but was operated in photovoltaic mode – current generated from incident light was amplified and measured with a picoammeter at each scanned wavelength step. The data acquisition program stepped the monochromator in 5 or 10 nm steps, accumulating 100 data samples at each wavelength step, reporting the average response and calculating the standard deviation of the 100 responses at each point.
